# The effect of heart rate on the heart rate variability response to autonomic interventions

**DOI:** 10.3389/fphys.2013.00222

**Published:** 2013-08-26

**Authors:** George E. Billman

**Affiliations:** Department of Physiology and Cell Biology, The Ohio State UniversityColumbus, OH, USA

**Keywords:** heart rate, heart rate variability, autonomic nervous system, cholinergic receptor antagonists, β-adrenergic receptors, exercise, baroreceptor reflex

## Abstract

Heart rate variability (HRV), the beat-to-beat variation in either heart rate (HR) or heart period (R-R interval), has become a popular clinical and investigational tool to quantify cardiac autonomic regulation. However, it is not widely appreciated that, due to the inverse curvilinear relationship between HR and R-R interval, HR *per se* can profoundly influence HRV. It is, therefore, critical to correct HRV for the prevailing HR particularly, as HR changes in response to autonomic neural activation or inhibition. The present study evaluated the effects of HR on the HRV response to autonomic interventions that either increased (submaximal exercise, *n* = 25 or baroreceptor reflex activation, *n* = 20) or reduced (pharmacological blockade: β-adrenergic receptor, muscarinic receptor antagonists alone and in combination, *n* = 25, or bilateral cervical vagotomy, *n* = 9) autonomic neural activity in a canine model. Both total (RR interval standard deviation, RRSD) and the high frequency (HF) variability (HF, 0.24–1.04 Hz) were determined before and in response to an autonomic intervention. All interventions that reduced or abolished cardiac parasympathetic regulation provoked large reductions in HRV even after HR correction [division by mean RRsec or (mean RRsec)^2^ for RRSD and HF, respectively] while interventions that reduced HR yielded mixed results. β-adrenergic receptor blockade reduced HRV (RRSD but not HF) while both RRSD and HF increased in response to increases in arterial blood (baroreceptor reflex activation) even after HR correction. These data suggest that the physiological basis for HRV is revealed after correction for prevailing HR and, further, that cardiac parasympathetic activity is responsible for a major portion of the HRV in the dog.

## Introduction

Heart rate variability (HRV, beat-to-beat changes in the heart period, R-R interval) is increasingly used to quantify cardiac autonomic regulation and to identify patients at an increased risk for adverse cardiovascular events (Appel et al., 1989; Task Force of the European Society of Cardiology, and the North American Society of Pacing and Electrophysiology, 1996; Berntson et al., 1997; Denver et al., 2007; Thayler et al., 2010; Billman, 2011, 2013). However, it is not widely appreciated that the prevailing heart rate (HR) can influence HRV independent of changes in cardiac autonomic regulation.

**Figure 1:**
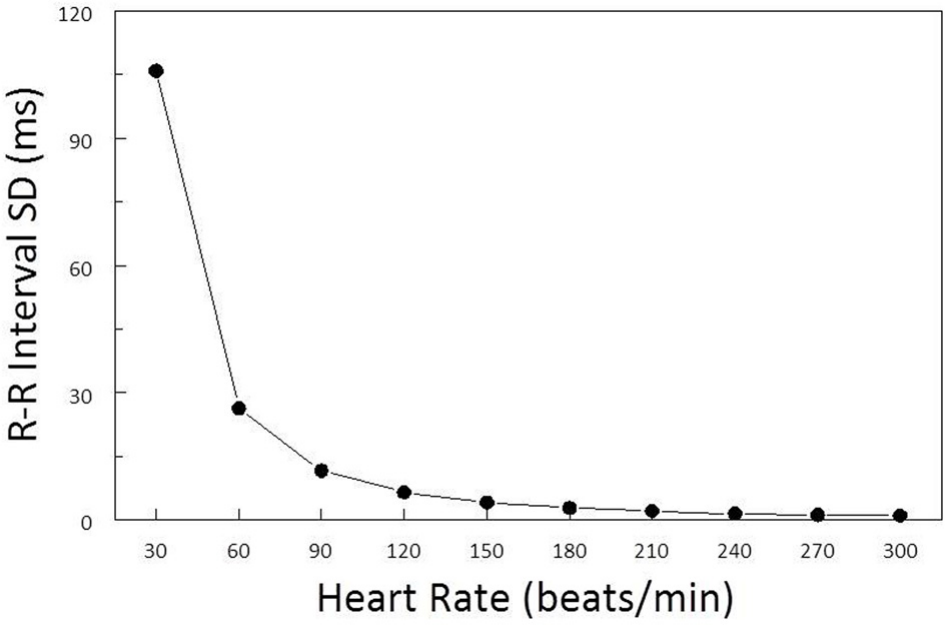
**Effect of baseline heart rate on heart rate variability.** The standard deviation of R-R interval (RRSD) was calculated for a set of 5 simulated heart beats (X − 2, X − 1, X, X + 1, X + 2) over a range of mean heart rates (HR, from 30 to 300 beats/min) (solid black line). The standard deviation for HR was ±1.6 beats/min at each HR level. Note that RRSD was inversely related to HR, identical changes in HR were accompanied by much larger R-R interval variability at low as compared to high prevailing HRs.

As a consequence of the inverse curvilinear relationship between HR and R-R interval, identical changes in HR will elicit profoundly different changes in the R-R interval variability depending upon the baseline HR (Sacha and Pluta, 2008). For examples, as is illustrated in Figure 1, the same HR variability (±1.6 beats/min) is associated with a much greater R-R interval variability (RRSD) at lower (RRSD at 30 beats/min = 105.9 ms) as compared to higher (RRSD at 180 beats/min = 2.9 ms) prevailing HRs. Several studies have reported a strong inverse correlation between HR and various time domain indices of HRV (e.g., the standard deviation (SD) of normal beats, SDNN; Kleiger et al., 1987; Van Hoogenhuyze et al., 1991; Fleiss et al., 1992) such that R-R interval variability increases as average HR decreases. Frequency domain analysis of HRV is similarly affected by mean HR. Sacha and co-workers (Sacha and Pluta, 2005a,b; Sacha et al., 2013a,b) demonstrated that the high frequency (HF) component of HRV was inversely, while the low frequency (LF) component was directly, related to average baseline HR of the subject. As such, differences in average HR *per se* will influence HRV magnitude independent of cardiac autonomic nerve activity either magnifying or masking (diminishing) the autonomic component of HRV as HR changes. It is therefore essential to correct HRV for the prevailing HR in order to identify physiological (changes in cardiac autonomic regulation), as opposed to artifactual (that merely arise as a consequence of a mathematical relationship), changes in HRV.

Although Sacha and co-worker (Sacha and Pluta, 2005a,b, 2008; Sacha et al., 2013a,b) have recently examined the relationship between average HR and indices of HRV under baseline conditions and compared methods to correct HRV for HR, the effects of HR on HRV during the activation or inhibition of cardiac autonomic regulation remained to be determined. As autonomic interventions will alter the prevailing HR, it is particularly important to correct indices of HRV for HR in order to differentiate between the HRV changes that are directly related to cardiac autonomic neural activation or inhibition from those changes that result merely as a mathematical consequence of increases or decreases in the baseline HR. It, therefore, was the purpose of the present study to evaluate the effects of well-characterized autonomic interventions on HRV after correction for average HR. Using a canine model, Cardiac autonomic neural activity was increased by submaximal exercise or the activation of the baroreceptor reflex and reduced by pharmacological (autonomic blockade: β-adrenergic receptor, muscarinic receptor antagonists alone and in combination) or by surgical (bilateral cervical vagotomy) interventions.

## Methods

All the animal procedures were approved by the Ohio State University Institutional Animal Care and Use Committee and conformed to the *Guide for the Care and Use of Laboratory Animals* published by the US National Institutes of Health (NIH publication N. 85-23, revised 1996).

Archived data from 74 heartworm free mixed breed dogs (1–3 y old, male *n* = 32, female *n* = 42) weighing 19.3 ± 0.4 kg (range = 11.6–26.8 kg) were used in the present study. The sole selection criterion was an ECG signal of sufficient quality to determine HRV both at baseline and in response to autonomic neural interventions (i.e., submaximal exercise, baroreceptor reflex activation, pharmacological autonomic blockade, or bilateral cervical vagotomy).

## Heart rate variability protocols

Body surface electrodes were placed on either side of the animal's chest and secured with surgical tape. HRV was then calculated using a Delta-Biometrics vagal tone monitor triggering off the electrocardiogram R-R interval (Urbana-Champaign, IL). This device employs the time-series signal processing techniques as developed by Porges to estimate the amplitude of respiratory sinus arrhythmia [the HF component of R-R interval variability (Porges, 1986)]. Details of this analysis have been described previously (Billman and Hoskins, 1989; Billman and Dujardin, 1990; Houle and Billman, 1999). Data were averaged over 30s intervals before and after the autonomic interventions (see below). The following indices of HRV were determined: Vagal Tone Index - the HF component of R-R interval variability (HF, 0.24–1.04 Hz), and SD of the R-R intervals (a marker of total variability) for the same 30 s time periods.

In order to remove any mathematical bias from HRV calculations, Sacha and co-workers (Sacha and Pluta, 2005a,b; Sacha et al., 2013a,b) previously demonstrated that SD of R-R and frequency data (power spectra) should be corrected by division with the corresponding mean R-R interval or mean R-R interval (in seconds) squared, respectively. These correction factors were used in all subsequent analyses.

## Autonomic interventions

Animals received the following interventions to increase or decrease cardiac autonomic regulation: pharmacological blockade (*n* = 25); baroreceptor reflex activation (*n* = 20); submaximal exercise (*n* = 25); and bilateral cervical vagotomy (*n* = 9).

### Autonomic blockade (*n* = 25)

First, the dogs were trained to lie quietly and unrestrained on a laboratory table. Once the animals had habituated to the laboratory environment, a catheter was percutaneously placed in a cephalic vein for the administration of a non-selective β-adrenergic receptor antagonist (propranolol HCl, 1.0 mg/kg, i.v.) followed, at least 5 min later, by a cholinergic muscarinic receptor antagonist (atropine sulfate, 50 μ g/kg, i.v.). The drug doses had been previously shown to provide an effective inhibition of cardiac autonomic neural receptors (Billman and Dujardin, 1990). One week later, the study was repeated with the drugs given in the reverse order (i.e., atropine followed by propranolol). HRV was monitored continuously 5 min before and for at least 5 min after each drug injection to ensure that peak changes had been achieved.

### Baroreceptor activation (*n* = 20)

With the animals lying quietly on a laboratory table, a bolus injection of the α-adrenergic receptor agonist, phenylephrine (10 μ g/kg, i.v.) was given to induce a 30–50 mm Hg increase in arterial pressure and thereby reflexively increase cardiac parasympathetic and decrease cardiac sympathetic neural activity (Billman et al., 1982). HRV was monitored for at least 5 min after the drug had been given to ensure that peak changes had occurred.

### Submaximal exercise (*n* = 25)

Over a period of 3–5 days, the dogs learned to run on a motor driven treadmill. The cardiac response to submaximal (i.e., 60–70% of maximal HR) exercise was then evaluated as follows: Exercise lasted a total of 18 minutes with workload increasing every 3-min. The protocol began with a 3-min “warm-up” period, during which the dogs ran at 4.8 kph at 0% grade. The speed was then increased to 6.4 kph, and the grade increased every 3-min (0, 4, 8, 12, and 16%). The submaximal exercise test was repeated three times (1/day). HRV was monitored continuously, beginning 3 min before the onset of exercise, during exercise, and for the first 3 min following the termination of exercise.

### Bilateral cervical vagotomy (*n* = 9)

Finally, as a terminal experiment, dogs were pre-medicated with morphine sulfate (2 mg/kg, i.m.). A catheter was percutaneously placed in a cephalic vein and used to administer the anesthesia: a mixture of α-chloralose (50 mg/kg, i.v.) and urethane (500 mg/kg, i.v). This anesthetic regimen has been shown to preserve cardiac autonomic regulation (Halliwill and Billman, 1992). The cervical vagus nerves were located via a midline incision on the ventral surface of the neck and 1 h later both vagus nerves were cut. HRV was once again monitored for at least 5 min after the nerves had been severed.

## Data analysis

All data are reported as mean SEM. The data were digitized (1 kHz) and recorded using a Biopac MP-100 data acquisition system (Biopac Systems, Inc., Goleta, CA). The HR and HRV data were averaged over 30 s intervals before and during the autonomic interventions.

All statistical analyses were performed using NCSS statistical software, (NCSS, Kaysville, UT). The relationship between baseline HR and HRV (SD of R-R interval or HF variability) with and without HR correction were evaluated by means of linear regression. The autonomic intervention data, with or without correction for HR, were compared using an ANOVA with repeated measures. Homogeneity of covariance (sphericity assumption, equal correlates between the treatments) was tested using Mauchley's test and, if appropriate, adjusted using Huynh–Feldt correction. If the *F*-value exceeded a critical value (*P* < 0.05), *post-hoc* comparisons of the data were then made using Tukey–Kramer Multiple-Comparison Test. The effect of anesthesia on baseline data was evaluated using a *t*-test.

## Results

### Relationship between baseline HR and HRV

**Figure 2:**
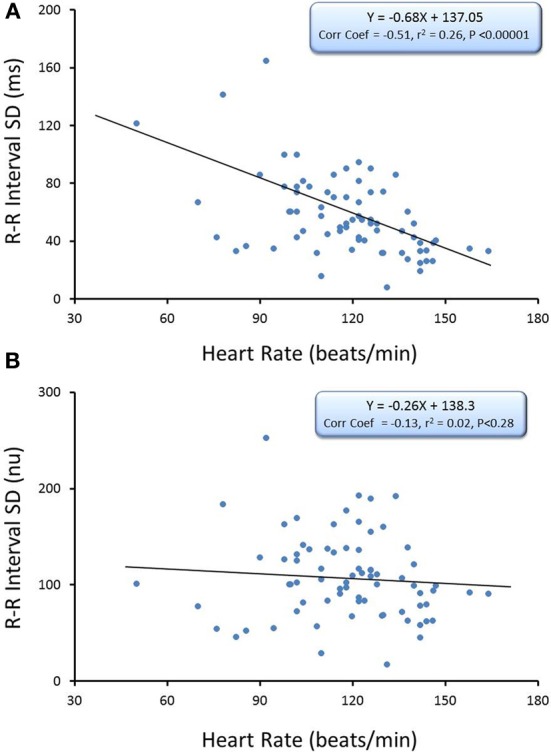
**The relationship between baseline heart rate and heart rate variability.** The total heart rate (HR) variability (standard deviation of R-R interval, RRSD) was calculated for over the last 30 s before an autonomic intervention was administered and plotted against the average HR for that interval. One data point is displayed for each animal (*n* = 74). The data without and with HR correction (RRSD/mean RR) are displayed in (**A,B)**, respectively. Note that HR only accounted for less than 30% (*r*^2^ = 0.26) of the variability before correction for HR. nu, normalized units following HR correction.

**Figure 3:**
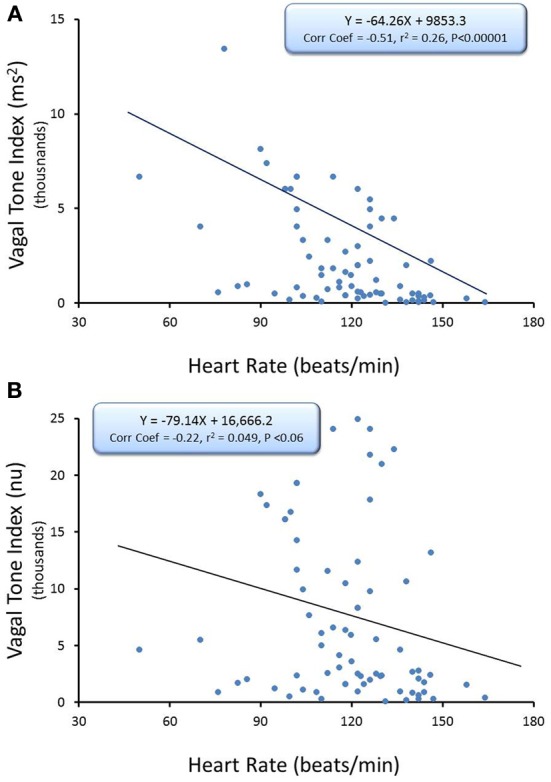
**The relationship between baseline heart rate and heart rate variability.** The high frequency (HF) component of the R-R interval variability (cardiac vagal tone index, 0.24–1.04 Hz) was calculated for over the last 30 s before an autonomic intervention was administered and plotted against the average HR for that interval. One data point is displayed for each animal (*n* = 74). The data without and with HR correction [cardiac vagal tone index/(mean RRsec)^2^] are displayed in **(A,B)**, respectively. Note that HR only accounted for less than 30% (*r*^2^ = 0.26) of the variability before correction for HR. nu, normalized units following HR correction.

The relationship between average HR and the R-R interval variability (SD of R-R interval, *n* = 74) and HF component of the R-R interval variability (cardiac vagal tone index, *n* = 74) under baseline conditions before and after correction for mean R-R interval are displayed in Figures 2, 3, respectively. There were significant inverse relationships between HR and either the R-R interval SD (RRSD, Pearson's correlation coefficient = −0.51, *P* < 0.00001; Figure 2A,) or the HF variability (Pearson's correlation coefficient = −0.51, *P* < 0.00001; Figure 3A). However, HR accounted for less than 30% of this variability (R-R variability, *r*^2^ = 0.26; HF variability *r*^2^ = 0.26). Correction for the prevailing HR abolished the HR dependence for both RRSD (Pearson's correlation coefficient = −0.128, NS; Figure 2B) and HF variability (Pearson's correlation coefficient = −0.221, NS; Figure 3B). The portion of this variability that could be ascribed to average HR was also eliminated after correction (R-R interval variability *r*^2^ = 0.0165; HF variability *r*^2^ = 0.0492).

### Pharmacological interventions—autonomic neural blockade

**Figure 4:**
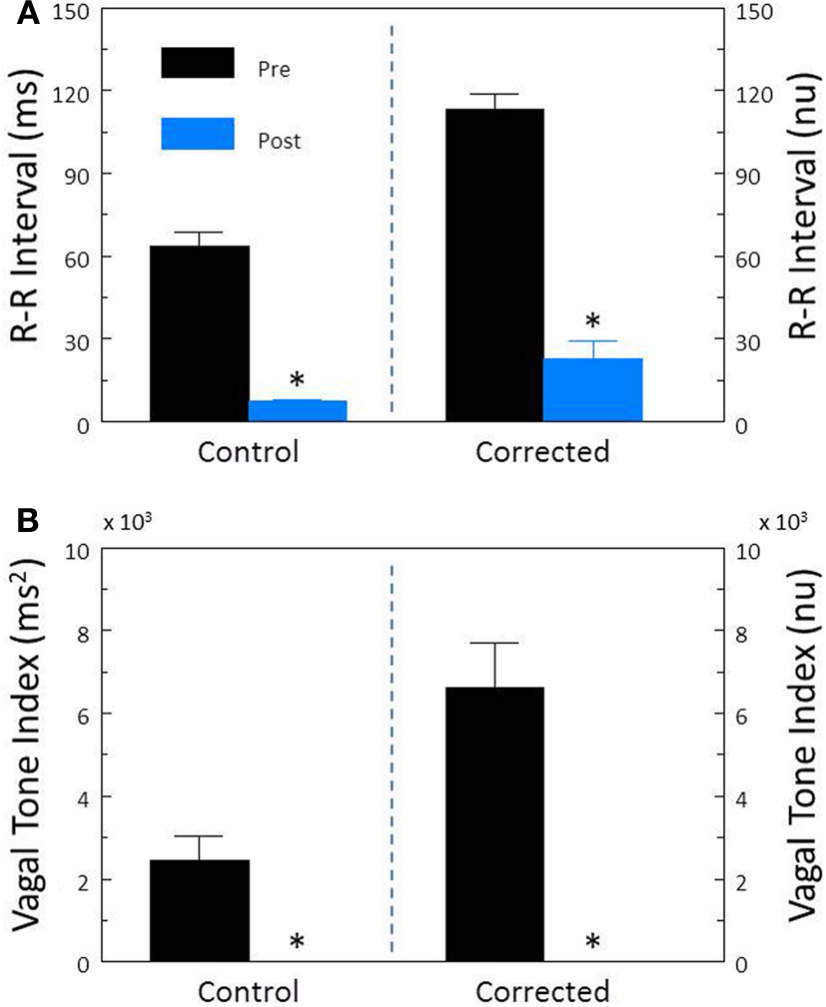
**The effect of the cholinergic receptor antagonist atropine on heart rate variability.** The effect of atropine sulfate (50 μ g/kg i.v.; *n* = 25) on total heart rate variability (standard deviation of R-R interval, RRSD) without and with correction (RRSD/mean RRsec) are displayed in **(A)**. The effects of this drug on the high frequency variability (cardiac vagal tone index, 0.24–1.04 Hz) without and with correction [cardiac vagal tone/(mean RRsec)^2^] are shown in **(B)**. Note that despite correction for large increases in heart rate, atropine still provoked large reductions in both RRSD and the cardiac vagal tone index. Thus, cardiac parasympathetic activity is responsible for a large portion of the heart rate variability independent of changes in HR. ^*^P < 0.01 pre (black bars) vs. post (blue bars); pre = last 30 s before atropine administration, post = 30 s interval recorded 5 min after atropine treatment. nu, normalized units following HR correction.

Cardiac parasympathetic regulation was inhibited using the cholinergic (muscarinic receptor) antagonist atropine sulfate. As would be expected, this drug elicited significant increases in HR (pre-atropine, 113.2 ± 4.7; post-atropine, 189.8 ± 5.2 beat/min, *P* < 10^−6^) and decreases in R-R interval (pre-atropine, 560.9 ± 33.6; post-atropine, 321.6 ± 8.5 ms, *P* < 10^−6^). Atropine treatment also provoked significant reductions (both *P* < 10^−6^) in R-R interval variability (Figure 4A) and HF variability (Figure 4B). After correction for prevailing HR, corrected R-R interval (Figure 4A) and corrected HF variability (Figure 4B) were still significantly (both *P* < 10^−5^) reduced by atropine treatment.

**Figure 5:**
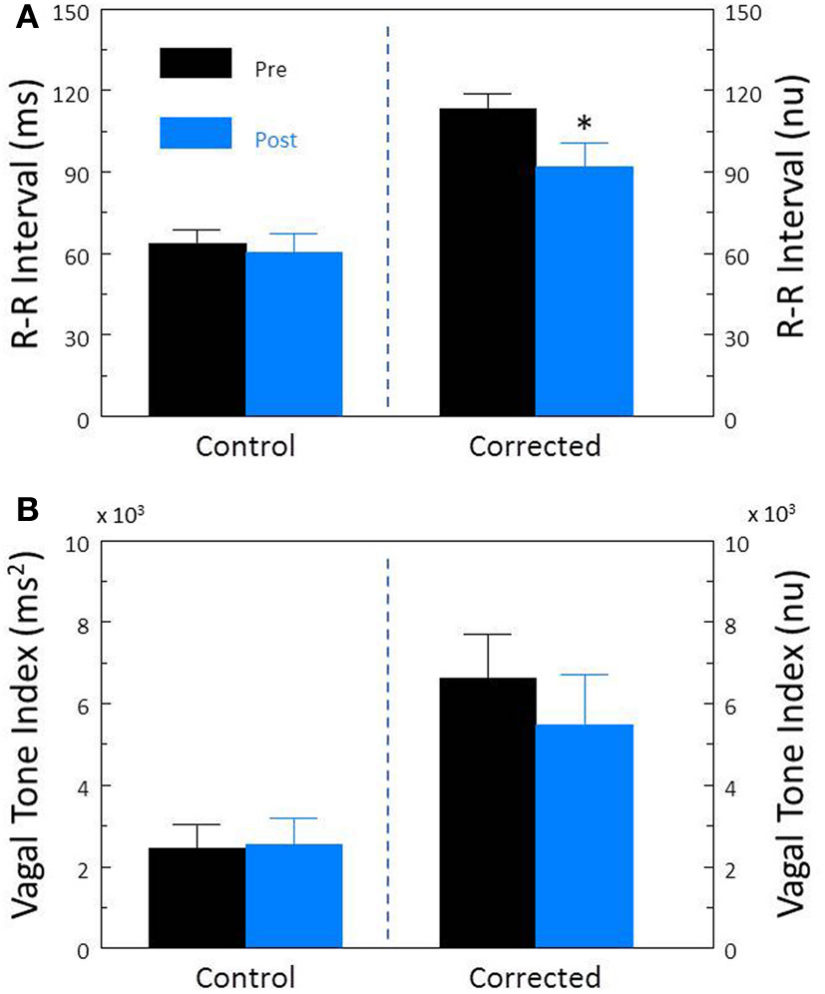
**The effect of the β-adrenergic receptor antagonist propranolol on heart rate variability.** The effect of propranolol HCl (1.0 mg/kg i.v.; *n* = 25) on total heart rate variability (standard deviation of R-R interval, RRSD) without and with correction (RRSD/mean RRsec) are displayed in **(A)**. The effects of this drug on the high frequency variability (cardiac vagal tone index, 0.24–1.04 Hz) without and with correction [cardiac vagal tone/(mean RRsec)^2^] are shown in **(B)**. Note that after correction for propranolol-induced reductions in baseline HR, total (RRSD) heart rate variability significantly decreased following this treatment. ^*^P < 0.01 pre (black bars) vs. post (blue bars); pre = last 30 s before propranolol administration, post = 30 s interval recorded 5 min after this drug treatment. nu, normalized units following HR correction.

In contrast, inhibition of cardiac sympathetic regulation using the non-selective β-adrenergic receptor antagonist propranolol HCl elicited significant reductions in HR (pre-propranolol, 114.0 ± 5.1; post-propranolol, 96.8 ± 2.6 beat/min, *P* < 0.002) and increases in R-R interval (pre-propranolol, 524.6 ± 26.6; post-propranolol, 631.6 ± 18.6 ms, *P* < 0.05). Propranolol treatment did not alter either R-R interval (*P* < 0.58, Figure 5A) or HF (*P* < 0.88, Figure 5B) variability before HR correction. However, after correction for the propranolol induced reductions in HR, this drug provoked significant reductions in corrected R-R interval variability (*P* < 0.007, Figure 5A) but not in corrected HF variability (*P* < 0.37, Figure 5B).

**Figure 6:**
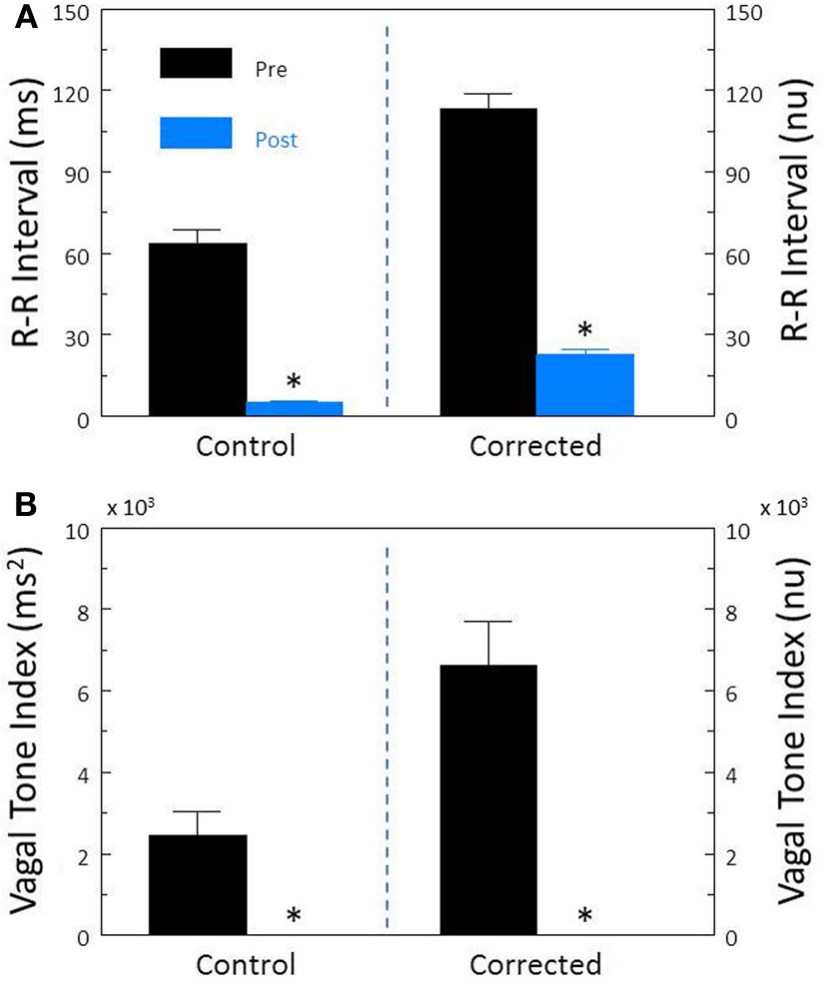
**The effect of total autonomic neural inhibition on heart rate variability.** Cardiac autonomic blockade (*n* = 25) was achieved using the combination of a cholinergic receptor antagonist (atropine sulfate, 50 μ g/kg i.v.) and a non-selective β-adrenergic receptor (propranolol HCl, 1.0 mg/kg i.v.). The effects of autonomic blockade on total heart rate variability (standard deviation of R-R interval, RRSD) without and with correction (RRSD/mean RRsec) are displayed in **(A)**. The effects of this treatment on the high frequency variability (cardiac vagal tone index, 0.24–1.04 Hz) without and with correction [cardiac vagal tone/(mean RRsec)^2^] are shown in **(B)**. Note that despite correction for large increases in heart rate, this treatment still provoked large reductions in both RRSD and the cardiac vagal tone index. As baseline HR increased following autonomic blockade, these data indicate the presence of a tonic parasympathetic restraint of intrinsic HR under basal conditions in the dog. ^*^P < 0.01 pre (black bars) vs. post (blue bars); pre = last 30 s before atropine + propranolol administration, post = 30 s interval recorded 5 min after this drug treatment. nu, normalized units following HR correction.

Complete autonomic blockade (atropine + propranolol) provoked significant increases in HR (pre-treatment, 113.2 ± 4.7; post-treatment, 149.3 ± 5.8 beats/min, *P* < 0.00007) and reductions in R-R interval (pre-treatment, 560.9 ± 33.6; post-treatment, 415.2 ± 14.6 ms, *P* < 0.0007). Autonomic blockade also provoked significant reductions in R-R interval variability (*P* < 10^−6^, Figure 6A) and HF variability (*P* < 0.0003, Figure 6B). After correction for HR, corrected R-R interval (Figure 6A) and corrected HF (Figure 6B) variability still significantly (both *P* < 10^−6^) decreased following complete autonomic blockade. As the post-autonomic blockade HR was higher than baseline HR, these data indicate the presence of a tonic parasympathetic regulation of HR under basal conditions in the dog.

### Physiological interventions—exercise or baroreceptor reflex activation

**Figure 7:**
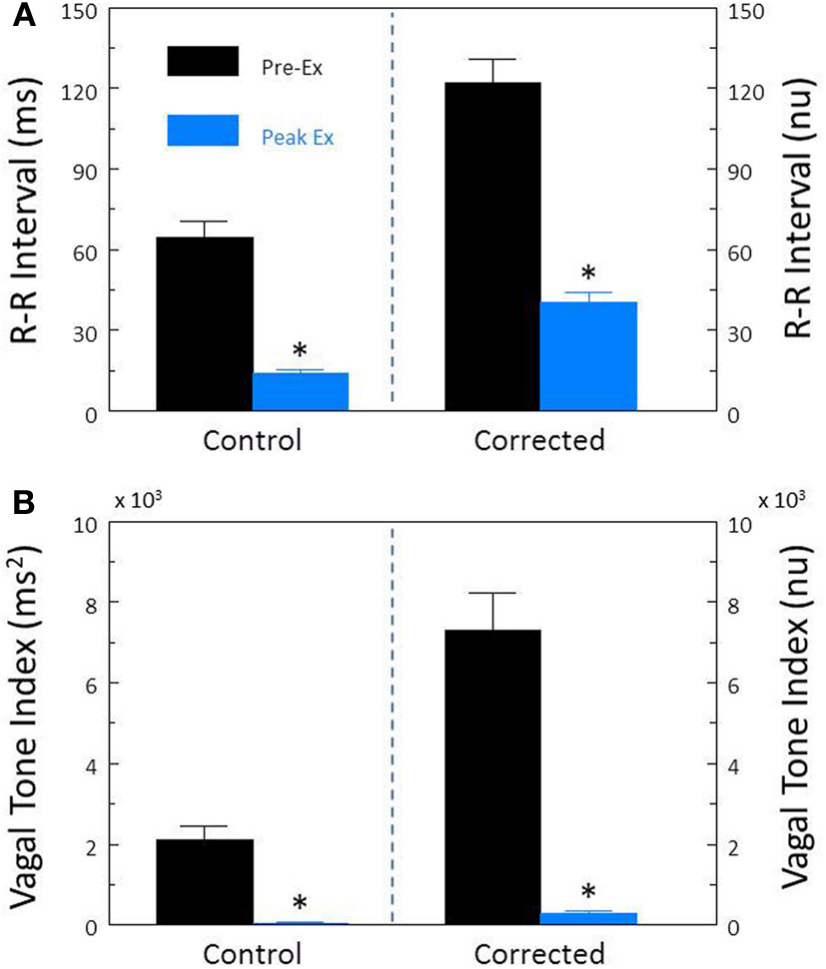
**Effect of submaximal exercise on heart rate variability.** The effect of exercise (*n* = 25) on total heart rate variability (standard deviation of R-R interval, RRSD) without and with correction (RRSD/mean RRsec) is displayed in **(A)**. The effect of exercise on the high frequency variability (cardiac vagal tone index, 0.24–1.04 Hz) without and with correction [cardiac vagal tone/(mean RRsec)^2^] are shown in **(B)**. Note that despite correction for large increases in heart rate, exercise provoked even large reductions in both RRSD and the cardiac vagal tone index than were noted before correction. The data were averaged over the last 30 s before exercise onset (Pre-Ex, black bars) and during the last 30 s of exercise (Peak Ex, blue bars) level. Peak exercise = 6.4 kph and 16% grade. ^*^P < 0.01 Pre-Ex vs. Peak Ex. nu, normalized units following HR correction.

In agreement with previous studies (Billman and Hoskins, 1989; Billman and Dujardin, 1990; Houle and Billman, 1999; Billman, 2006, 2009) exercise elicited significant increases in HR (pre-exercise, 119.5 ± 3.8; peak-exercise, 181.7 ± 4.7 beats/min, *P* < 10^−6^) and reductions in R-R interval (pre-exercise, 514.2 ± 16.2; peak-exercise, 336.1 ± 10.0 ms, *P* < 10^−6^) that were accompanied by significant (*P* < 10^−6^) reductions in both R-R interval (Figure 7A) and HF (Figure 7B) variability. After correction for the prevailing HR, exercise still provoked large reductions in both the corrected R-R variability (*P* < 10^−6^, Figure 7A) and the corrected HF variability (*P* < 10^−6^, Figure 7B; both).

**Figure 8:**
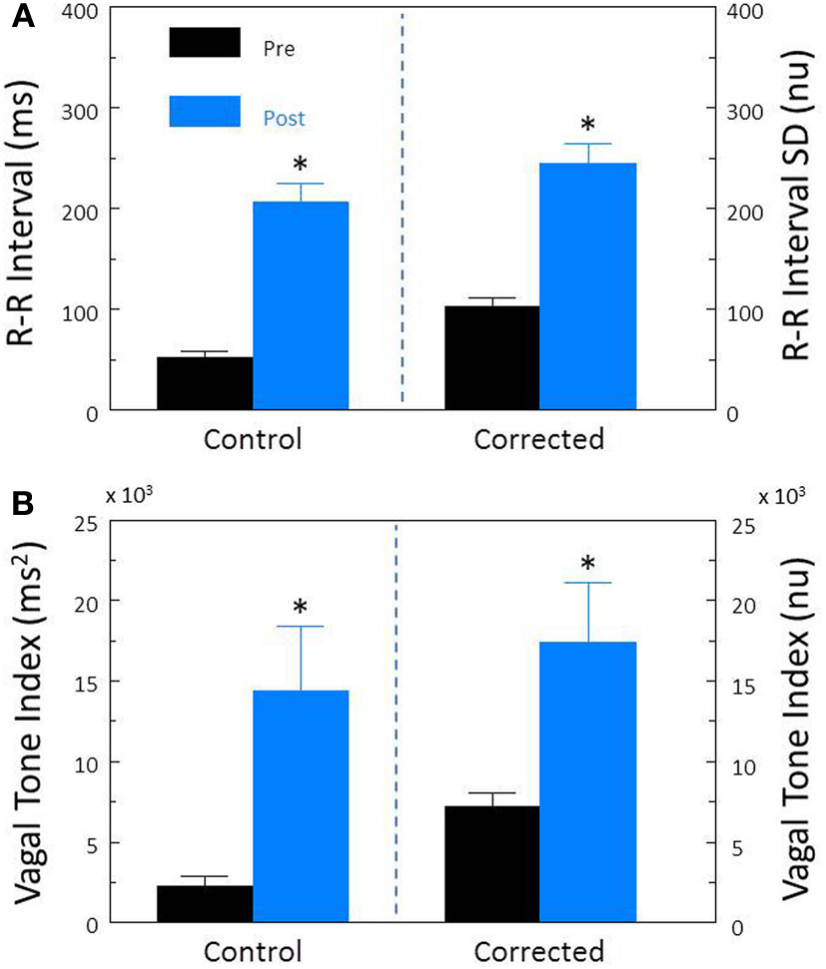
**The effect of activation of the baroreceptor reflex on heart rate variability.** The α-adrenergic agonist phenylephrine HCl (10 μ g/kg, i.v; *n* = 20) was used to increase arterial blood 30–50 mm Hg and thereby reflexively induce reductions in heart rate. The effects of this intervention on total heart rate variability (standard deviation of R-R interval, RRSD) without and with correction (RRSD/mean RRsec) are displayed in **(A)**. The effects of this treatment on the high frequency variability (cardiac vagal tone index, 0.24–1.04 Hz) without and with correction [cardiac vagal tone/(mean RRsec)^2^] are shown in **(B)**. Note that both before and after correction for the phenylephrine-induced decreases in HR, baroreceptor reflex activation provoked significant increases in both RRSD and the cardiac vagal tone index. These data suggest that a reflexively mediated increase in cardiac parasympathetic activity is responsible for a large portion of the heart rate variability response to increases in arterial pressure independent of changes in HR. ^*^P < 0.01 pre (black bars) vs. post (blue bars); pre = last 30 s before phenylephrine administration, post = 30 s interval recorded 5 min after this physiological intervention. nu, normalized units following HR correction.

The α-adrenergic receptor agonist, phenylephrine (PE) was used to increase arterial pressure (via vasoconstriction) and thereby reflexively augmented cardiac parasympathetic and reduced cardiac sympathetic neural activity (baroreceptor reflex activation). In agreement with previous studies (Billman et al., 1982; Billman and Dujardin, 1990), phenylephrine provoked significant decreases in HR (pre-PE, 122 ± 5.0; PE, 74.3 ± 4.0 beats/min, *P* < 10^−6^) and increases in R-R interval (pre-PE, 507.3 ± 23.5; PE, 864.6 ± 58.1 ms, *P* < 10^−5^) that were accompanied by significant (both *P* < 10^−6^) increases in R-R interval (Figure 8A) and HF (Figure 8B) variability. After correction for the PE induced reductions in HR, baroreceptor activation produced similar increases in both corrected R-R interval (*P* < 10^−5^, Figure 8A) and corrected HF (*P* < 10^−6^, Figure 8B).

### Surgical intervention—bilateral cervical vagotomy

In contrast to previous reports (Halliwill and Billman, 1992), anesthesia reduced baseline HRV. Although baseline HR was not affected by anesthesia (conscious 113.2 ± 4.7 vs. anesthesia 110.8 ± 7.4 beats/min; *P* < 0.34), both RRSD (conscious, 63.7 ± 5.0 vs. anesthesia, 34.2 ± 4.4 ms; *P* < 0.001) and HF variability (conscious, 6622.3 ± 1089.5 vs. anesthesia, 3090.6 ± 1382.8 ms^2^, *P* < 0.05) were significantly lower in anesthetized (*n* = 9) as compared to conscious (*n* = 33) dogs. These differences in HRV were not altered by HR correction. Thus, anesthetic agents that were believed to have minimal effects on cardiac autonomic regulation (Halliwill and Billman, 1992) reduced HRV in the present study.

**Figure 9:**
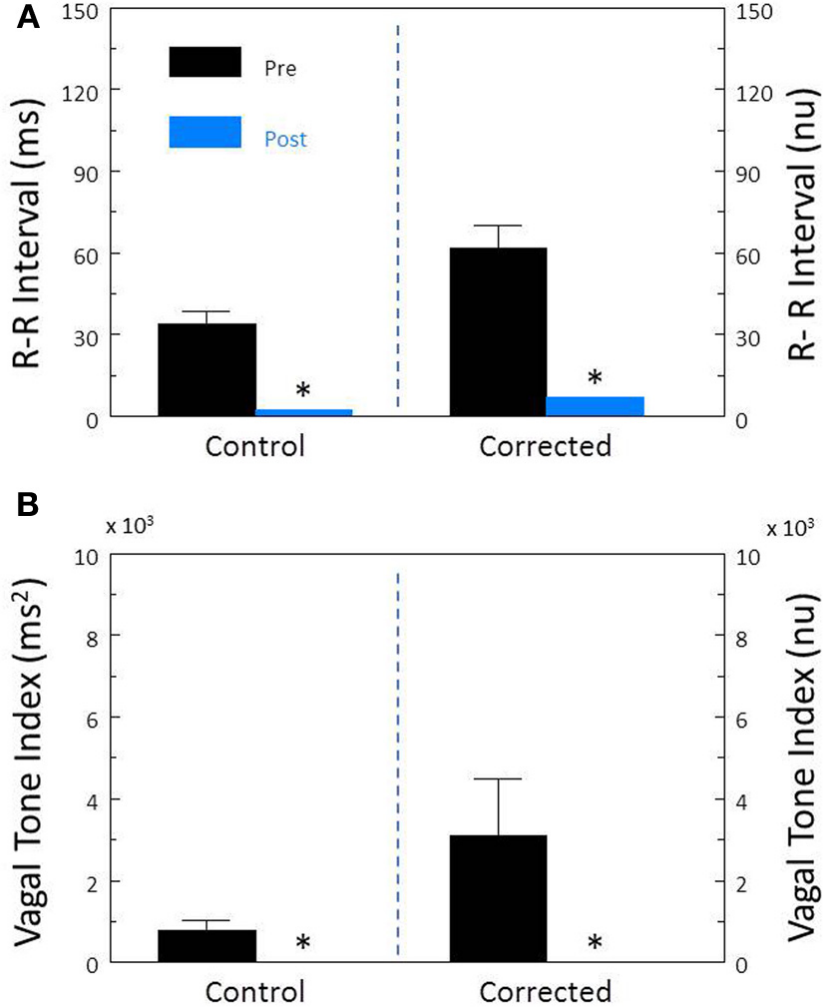
**The effect of bilateral cervical vagotomy on heart rate variability.** The effect of surgical disruption of the vagus nerves (*n* = 9) on total heart rate variability (standard deviation of R-R interval, RRSD) without and with correction (RRSD/mean RRsec) are displayed in **(A)**. The effects of this interval on the high frequency variability (cardiac vagal tone index, 0.24–1.04 Hz) without and with correction [cardiac vagal tone/(mean RRsec)^2^] are shown in **(B)**. Note that despite correction for large increases in heart rate, the vagotomy still provoked large reductions in both RRSD and the cardiac vagal tone index. These changes are very similar to those noted after treatment with the cholinergic receptor antagonist atropine. Thus, the vagotomy data further indicate that cardiac parasympathetic activity is responsible for a large portion of the heart rate variability independent of changes in HR ^*^P < 0.01 pre (black bars) vs. post (blue bars); pre = last 30 s before vagotomy, post = 30 s interval recorded 5 min after this treatment. nu, normalized units following HR correction.

Disruption of the cardiac parasympathetic regulation by bilateral cervical vagotomy elicited significant increases in HR (pre-vagotomy, 110.2 ± 7.4; post-vagotomy, 186.0 ± 9.8 beat/min, *P* < 0.00008) and decreases in R-R interval (pre-vagotomy, 561.3 ± 37.5; post-vagotomy, 329.7 ± 17.0 ms, *P* < 0.00004) that were accompanied by significant reductions in both R-R interval (*P* < 0.00009, Figure 9A) and HF (*P* < 0.0007, Figure 9B) variability. After correction for HR, vagotomy still produced significant reductions in both corrected R-R interval (*P* < 0.0002, Figure 9A) and corrected HF variability (*P* < 0.05, Figure 9B). These results are very similar to those obtained following treatment with atropine sulfate and further demonstrate that cardiac parasympathetic activity is responsible for a major portion of the HRV, independent of changes in the prevailing HR.

## Discussion

The present study investigated the effects of well-characterized autonomic interventions on HRV with and without correction for the prevailing HR. The major findings of the study are as follows: (1) In agreement with previous studies (Kleiger et al., 1987; Van Hoogenhuyze et al., 1991; Fleiss et al., 1992; Sacha and Pluta, 2005a,b, 2008; Sacha et al., 2013a,b), there were significant inverse relationships between HR and both total variability (R-R interval SD) and the variability within the HF band (0.24–1.04 Hz), an indirect marker of cardiac parasympathetic regulation (Appel et al., 1989; Task Force of the European Society of Cardiology, and the North American Society of Pacing and Electrophysiology, 1996; Berntson et al., 1997; Denver et al., 2007; Thayler et al., 2010; Billman, 2011). However, HR accounted for less than 30% of the HRV; (2) division of the HRV indices by the mean R-R (reported in seconds) or mean R-R interval (in seconds) squared (Sacha and Pluta, 2005b) successfully removed variability due to HR (Figures 2, 3) under basal conditions.; (3) Surgical (bilateral cervical vagotomy), pharmacological (cholinergic or complete autonomic blockade), and physiological (submaximal exercise) interventions that reduced or abolished cardiac parasympathetic regulation provoked large reductions in HRV even after correction for the accompanying increases in mean HR that were induced by these interventions; and (4) interventions that reduced HR yielded mixed results. β-adrenergic receptor blockade (propranolol) reduced rather than increased R-R interval variability after correction for the drug-induced HR reductions while, in contrast, increases in arterial blood pressure still provoked increases in both HF and R-R interval variability even after correction for the reflexively mediated reductions in HR. When considered together these data suggest that the physiological basis for HRV is revealed after correction for prevailing HR. These data further demonstrate that cardiac parasympathetic activity is responsible for a major portion of the HRV independent of changes in the prevailing HR and further that cardiac parasympathetic regulation provides a tonic restraint (inhibition) of the baseline pacemaker rate (i.e., the presence of a high basal vagal tone) in the dog.

As was previously noted, due to mathematical considerations, identical changes in HR can elicit profoundly different changes in R-R interval variability depending on the prevailing HR (larger changes at low, as compared to high, basal HR heart rates) independent of changes in cardiac neural activity (Sacha and Pluta, 2008). For example, a simulated set of heart beats with the same HR variability (*SD* = ±1.6 beats/min) at each HR level yielded markedly different values for R-R variability depending upon the prevailing HR (*HR* = 30 beats/min, RRSD = 105.9 ms vs. *HR* = 300 beats/min, RRSD = 1.1 ms; Figure 1). Similar, albeit less dramatic, results have been reported for data obtained from healthy subjects and in patients following myocardial infarction or with congestive heart disease (Kleiger et al., 1987; Van Hoogenhuyze et al., 1991; Fleiss et al., 1992). Indeed, a strong inverse correlation between HR and various time domain indices of HRV (e.g., the SD of normal beats, SDNN) was reported in these patient populations. Frequency domain analysis of HRV is similarly affected by mean HR. Sacha and co-workers (Sacha and Pluta, 2005a,b, 2008; Sacha et al., 2013a,b) demonstrated that the HF component of HRV was inversely, while the LF component was directly, related to average baseline HR of the subject. In agreement with these studies, similar results were obtained for the dog in the present study. Under basal conditions, both total variability (R-R interval SD) and the variability within the HF band (0.24–1.04 Hz) increased as HR decreased. However, only about 30% of this variability could be attributed to HR, demonstrating that other factors must also contribute to this variability. Thus, it is critical to remove the HR contribution from indices of the HRV in order to identify any physiological components to this variability. This HR correction is particularly important when cardiac autonomic neural regulation is altered, as the activation or inhibition of these cardiac nerves will produce corresponding changes in the prevailing HR, thereby making it difficult to discern the direct autonomic neural contribution to HRV under these conditions.

Recently, Sacha and co-workers (Sacha and Pluta, 2005b; Sacha et al., 2013a,b) demonstrated that division by corrections factors weakened the HR dependence of HRV. In particular, they found that the mathematical contribution to R-R interval (RRSD) and HF variability could be removed by dividing these variables by the corresponding mean R-R interval and (mean R-R)^2^, respectively. These earlier observations in human subjects were confirmed for healthy dogs in the present study, as these correction factors eliminated the correlation with prevailing HR under basal conditions (Figures 2, 3). Using these correction procedures, it was then possible to evaluate the effects of autonomic interventions on HRV that arise independent of changes in HR.

Interventions that reduce cardiac parasympathetic activation provoke HR increases and could, thereby, exaggerate the resulting reductions in HRV. In the present study, both pharmacological and surgical disruption of the cardiac parasympathetic nerves produced similar increases in HR and reductions in HRV. Exercise [a physiological challenge known to decrease cardiac parasympathetic and increase cardiac sympathetic activity (Billman, 2009)] also provoked large increases in HR that were accompanied by decreases in HRV. However, the HRV response to these interventions was not altered by correction for prevailing HR. These data strongly suggested that, even after correction for HR, cardiac parasympathetic regulation was responsible for a major portion of the reduction in indices of HRV provoked by these interventions. In a similar manner, the HRV reductions that resulted from complete autonomic blockade were not altered by correction for HR. As the prevailing HR increased following this treatment, these data further suggest that cardiac parasympathetic regulation provides a tonic inhibition of the basal HR in the dog.

In contrast to interventions that increased HR, autonomic interventions that reduced HR yielded mixed results. β-adrenergic receptor blockade (propranolol) did not alter HRV despite reductions in HR. However, after correction for drug-induced HR reductions, total (RRSD), but not HF, variability significantly decreased. For mathematical reasons, as previously discussed, one would expect that any intervention that decreases HR would produce increases in HRV. Thus, it is initially surprising that HF variability did not change and R-R interval variability decreased rather than increased as the result of propranolol treatment. There are at least two possible explanations for these observations: (1) Sympathetic neural activity could modulate the HF component of the R-R interval variability (Taylor et al., 2001; Cohen and Taylor, 2002). Taylor et al. (2001) found that cardioselective β-adrenergic receptor blockade increased the amplitude of respiratory sinus arrhythmia [a widely used marker of cardiac parasympathetic activity (Billman, 2011)] over a wide range of respiratory frequencies (i.e., the increases were not restricted to lower frequencies, <0.15 Hz). Thus, they concluded that “*cardiac sympathetic outflow can oppose vagally mediated R-R interval oscillations and sympathetic blockade removes this effect*” (Cohen and Taylor, 2002). However, since β-adrenergic receptor blockade decreased rather than increased R-R interval variability and did not alter HF variability in the present study, it is unlikely that the removal of a sympathetic restraint on cardiac vagal regulation can explain this observation. Indeed, it is possible that the HRV increases reported by Taylor and associates following β-adrenergic receptor blockade resulted as a mathematical consequence of declining HR rather than from the removal of any sympathetic “restraint” of cardiac parasympathetic regulation. (2) It is much more likely that inhibition of the cardiac sympathetic activity provoked the withdrawal of cardiac parasympathetic activity in order to maintain a more constant cardiac output. If this hypothesis is correct, then would one predict that this putative parasympathetic withdrawal should become more obvious during physiological challenges that increase tissue oxygen demand (that must be matched by increased oxygen delivery). In fact, both submaximal exercise and acute myocardial ischemia provoked much larger reductions in HF variability, despite smaller increases in HR, following β-adrenergic receptor blockade (Billman and Hoskins, 1989; Collins and Billman, 1989; Billman and Dujardin, 1990; Billman, 2006). Finally, and in contrast to β-adrenergic receptor blockade, the increase in HRV (both RRSD and HF) elicited in response to the increases in arterial blood pressure (induced by phenylephrine) was not altered after correction for the reflexively mediated reductions in HR. These data further suggest that an augmentation of cardiac parasympathetic activity can increase HRV independent of reflex mediated reductions in HR.

In conclusion, the present study demonstrates that prevailing HR can dramatically affect HRV with HRV increasing as HR decreases. The HR dependence of HRV becomes particularly important as HR changes in response to the activation or inhibition of cardiac autonomic neural regulation. It is, therefore, essential to correct HRV for the average HR in order to differentiate between physiologically- and mathematically- mediated changes in HRV. For the dog, as has been previous shown for human subjects (Sacha and Pluta, 2005b), R-R (as measured by SD) and HF variability can be corrected by division by mean R-R interval (in seconds) and (mean R-R interval, in seconds)^2^, respectively. HR correction did not attenuate the HRV response to interventions that inhibited cardiac parasympathetic regulation. As such, these data demonstrate that cardiac parasympathetic activity is responsible for a major portion of the HRV independent of changes in the prevailing HR. In contrast, interventions that reduced HR yielded mixed results after HR corrections. β-adrenergic receptor blockade decreased rather than increased some HRV indices after correction for HR suggesting that this treatment provoked compensatory reductions in cardiac parasympathetic activity (to maintain a more constant cardiac output in the face of changing environmental demands). In contrast, even after correction for baroreceptor reflex mediated reductions in HR, increases in arterial pressure still provoked large increases in HRV (both RRSD and HF variability). These data suggest that baroreceptor reflex mediated increases in are HRV largely result from the direct cardiac actions of parasympathetic activation. When considered together, these data are further evidence that HRV provides an indirect and largely qualitative assessment of cardiac parasympathetic regulation; an assessment that must also be corrected for prevailing HR. Since an accurate assessment of nerve activity can only be obtained from direct nerve recordings, HRV data should always be interpreted with care.

### Conflict of interest statement

The author declares that the research was conducted in the absence of any commercial or financial relationships that could be construed as a potential conflict of interest.
